# Overview of BioCreAtIvE task 1B: normalized gene lists

**DOI:** 10.1186/1471-2105-6-S1-S11

**Published:** 2005-05-24

**Authors:** Lynette Hirschman, Marc Colosimo, Alexander Morgan, Alexander Yeh

**Affiliations:** 1 The MITRE Corporation, 202 Burlington Road, Bedford, MA 01730, USA

## Abstract

**Background:**

Our goal in BioCreAtIve has been to assess the state of the art in text mining, with emphasis on applications that reflect real biological applications, e.g., the curation process for model organism databases. This paper summarizes the BioCreAtIvE task 1B, the "Normalized Gene List" task, which was inspired by the gene list supplied for each curated paper in a model organism database. The task was to produce the correct list of unique gene identifiers for the genes and gene products mentioned in sets of abstracts from three model organisms (Yeast, Fly, and Mouse).

**Results:**

Eight groups fielded systems for three data sets (Yeast, Fly, and Mouse). For Yeast, the top scoring system (out of 15) achieved 0.92 F-measure (harmonic mean of precision and recall); for Mouse and Fly, the task was more difficult, due to larger numbers of genes, more ambiguity in the gene naming conventions (particularly for Fly), and complex gene names (for Mouse). For Fly, the top F-measure was 0.82 out of 11 systems and for Mouse, it was 0.79 out of 16 systems.

**Conclusion:**

This assessment demonstrates that multiple groups were able to perform a real biological task across a range of organisms. The performance was dependent on the organism, and specifically on the naming conventions associated with each organism. These results hold out promise that the technology can provide partial automation of the curation process in the near future.

## Background

**Figure 1:**
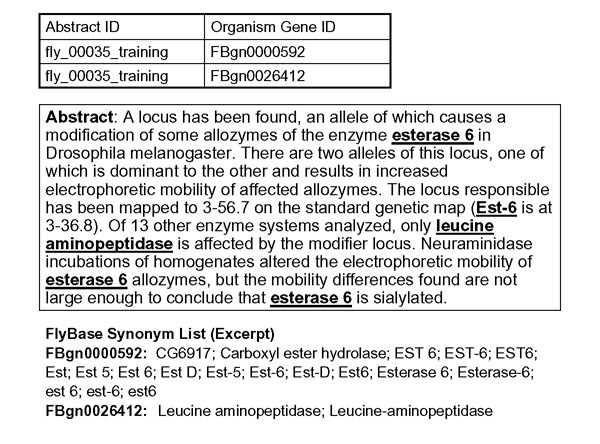
Abstract with gene list and synonym list excerpt.

Task 1B, the normalized gene list task, is intermediate in the BioCreAtIvE tasks. It builds on task 1A, the gene mention identification task [1], but it is much simpler and requires far less understanding of the underlying biology than task 2, functional annotation from text [2]. It reflects a step in the curation process for the model organism databases: once an article is selected for curation, an important step is to list those genes discussed in the article that have sufficient experimental evidence to merit curation – see discussion in [3]. Therefore, we were able to extract the expert-curated gene lists from the model organism databases, to use as training and test data. We chose to use Fly [4], Mouse [5], and Yeast [6] model organism databases as our three sources of data. Figure 1 shows a sample abstract from MEDLINE, together with the gene list for that abstract (top) from FlyBase.

Evaluation for task 1B is straightforward: it consists of comparing lists of unique identifiers. This makes it much easier to evaluate than the other tasks in BioCreAtIvE. Task 1A required the comparison of annotated text segments, raising issues of how to annotate complex gene names (e.g., *TTF-1-binding sites (TBE) 1, 3, and 4*), as well as questions about gene name boundaries. Task 2 required expert human evaluation of whether a text passage constitutes adequate evidence for a particular Gene Ontology annotation. Originally, for task 1B, we had also wanted evidence for each answer, parallel to the evidence passages required for task 2, but our instructions for this were not clear, different people submitted different things and we did not evaluate this.

In order to make the task uniform across the different model organisms and easily accessible to non-biologists, we extracted synonym lists from each of the three model organism databases. For each organism, the synonym list consisted of the list of unique gene identifiers and their associated gene symbol and synonyms. We made these lists available in a simple standard flat file format. Figure 1 (bottom) shows two entries from the synonym list for Fly. By providing a uniform set of lexical resources for each model organism, we hoped to encourage experimentation with techniques that could readily generalize to new organisms. However, participants were also allowed to use additional lexical resources, and a several groups took advantage of this.

We chose to use abstracts as the basis for the gene list task, rather than full text articles. This simplified the task for the participants, since abstracts are much shorter and easier to process than full text article (because they are around 250 words long and are available in ASCII). The abstracts can also be readily collected and distributed to the participants, unlike the full text articles. However, using abstracts meant that we had to prune the gene lists provided by the model organism database, since these were usually based on the full text articles. Table 1 shows the size of the training, development test and blind test data sets. To prepare the training data, we developed an automated pruning procedure to remove genes from the gene list that were not mentioned in the abstract. As discussed in [3], this was a "noisy" process. We delivered the noisy training data "as is" but we hand corrected the development test data and the blind test data. In later experiments, we estimated the quality of the noisy training data for Yeast at an F-measure of 92% (Table 2, 2^nd ^row); for Fly it was 83% (Table 3, 2^nd ^row); and for Mouse, it was 71% (Table 4, 2^nd ^row).

In addition to pruning the gene lists to reflect the content in the abstracts, we made one additional simplification in the task. The model organism databases do not curate every gene mentioned in a paper – they curate only those genes that meet a set of (organism-specific) criteria, including presentation of experimental evidence related to gene or gene included in the gene list. However, we felt that the abstract might not provide enough context to determine whether a gene had sufficient evidence for curation or was mentioned only in passing, so for the test data sets, the annotators added, by hand, all genes mentioned in the abstract. This was not done for the automatically generated training data, so the automatically generated training set had significant recall errors (see Tables 2, 3, 4).

## Results

Tables 2, 3, 4 show the scores from each participating system, by group and run (each run was considered a system) for Yeast (Table 2), Fly (Table 3) and Mouse (Table 4). Each group was allowed to submit up to three systems for each organism. The systems were scored against the manually created "gold standard" for each abstract in the test set (250 abstracts per organism). The results are presented in terms of the following metrics:

True Positives: Number of correctly detected genes

False Positives: Number of genes incorrectly marked as being present

Misses: Number of genes NOT detected by the system

Precision: True Positives / (True Positives + False Positives)

Recall: True Positives/ (True Positives + Misses)

F-measure: Balanced precision/recall computed as 2*P*R/(P+R)

The first two rows of each table show first the **Gold Standard **compared to itself, which always yields a score of 100% or 1. The second line, **Noisy Training**, shows the results of comparing the test data run through the "automatic cleaning" procedure and compared to the Gold Standard. This provides an estimate of the quality of the automatically generated training data.

Next, for each organism, we show High, Median and Low scores for each of these quantities, followed by the scores of each group by run.

**Figure 2:**
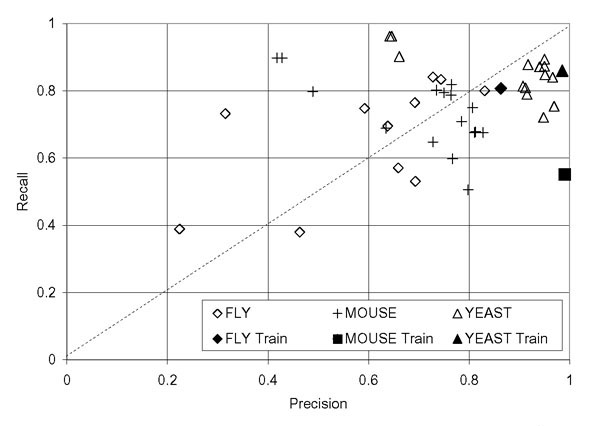
Task 1B results for all organisms: precision vs. recall.

In addition to the tables, Figure 2 shows a composite graph of precision versus recall for all systems and all organisms. This graph also shows the estimates of training data quality (marked as Yeast Train, Fly Train and MouseTrain in the legend and in solid symbols on the graph). The diagonal line indicates balanced precision versus recall.

The results demonstrate several things, in particular, that there are significant differences among organisms.

1. Yeast is the easiest. The F-measures of the systems tended to be high, with several groups achieving an F-measure of over 0.90, and a median F-measure of 0.86. Also, the quality of the training data was high (F-measure 0.92).

2. Fly was harder than Yeast: the high F-measure was 0.82, and there was much greater variability in performance (median F-measure was 0.66). The training data quality for Fly was significantly lower than for Yeast (0.83). Fly was hard because there are many ambiguous terms, and there is also extensive overlap between Fly gene name abbreviations and English words, such as "not", "period", "was", etc.

3. Mouse was the hardest as measured by system performance (best F-measure 0.79), although the median system performance for Mouse was better than for Fly (0.74). The training data quality was the lowest (F-measure of 0.71), with a high precision (99%) but a low recall (55%). The poor training data quality was related to the stringent Mouse curation criteria. Because of this, there were relatively many more genes that were mentioned in the article but not judged to be appropriate for curation (and therefore, not on the list of curated genes from the MGI database). These genes were not included in the automatically generated training data, hence the low recall and low F-measure for the training data. Of course, such mentions were added manually into the development test data and blind test data. Indeed, for Mouse, the median system F-measure was actually higher than the training data F-measure, indicating that the systems did a good job in generalizing away from the noise.

A second observation is that systems may have been limited by the quality of the noisy training data. For both Yeast and Fly, the estimated training data quality was just a shade higher than the final top performing systems.

## Methods

This section discusses the methods used to prepare the evaluation materials.

### Data preparation

In order to evaluate the performance of the systems, the organizers prepared a hand-coded gold standard, as described in [3]. First, each abstract was associated with the gene ID list from the appropriate model organism database. Since we were using abstracts rather than full text, the gene list from the model organism database then had to be adjusted to conform to the names mentioned in the abstract. This was done in several steps, as follows:

• Removing gene IDs that were not found in the abstract, but were found in the underlying full text article. This was done automatically, using the synonym list, to generate large quantities of "noisy" training data. This corresponds to the **Noisy Training **column on the tables for the model organism performance data.

• Hand checking to make sure that the automatic procedure did not eliminate genes that were present in the abstract (development test set and blind test set only). This could occur if, for example, the mention in the text was a variant of the synonyms provided in the lexical resource, e.g., "polgamma B" versus "polgamma 2".

• Adding in any additional genes mentioned "in passing" in the abstract (development test set and blind test set only). This was necessary because each model organism database curates genes according to a certain set of criteria, so not all genes mentioned are necessarily on the gene list. There might, for example, be additional genes mentioned "in passing," such as genes located near a gene of interest, or possible homologues etc.

Overall, we estimate that it took between 1–2 staff weeks of time from an experienced curator to edit and check a 250 abstract test set. The checking was particularly important because we detected significant interannotator variability, particularly for the Mouse annotations – see [3] for a detailed discussion of the data preparation and interannotation agreement studies.

### Lexical resources

An analysis of the lexical resources provides insight into the differences in difficulty observed for the three organisms. Table 5 gives a picture of the amount of synonymy in the different lexicons. It shows the number of unique identifiers (IDs), the number of terms in the lexicon, and the average number of terms per identifier (synonyms) for each organism. We can see that the Yeast resources are the most parsimonious (1.9 synonyms per ID). Fly is the richest with 2.9 synonyms per ID, but note also the large standard deviation of 3.9: 42% of Fly identifiers have only one term and only 15% have more than 4 synonyms per ID. In addition, the last column of Table 5 shows the average length (in words) for the terms. Again, Yeast is very compact, with barely over one word per term; this almost certainly contributed to the high performance on Yeast. Mouse has the longest terms on average, at 2.77 words per synonym, but with a large standard deviation (2.6) Overall, 58% of Mouse terms were one word long and 81% of the terms were four words long or less. The complexity of the Mouse terms (as measured by length) may have contributed to recall problems in identifying gene mentions, since longer names tend to be more descriptive and therefore, to show significant syntactic variation. Also, the task 1A results [1] indicate that longer names are more difficult to identify.

The resources for these organisms also differ in amount of ambiguity among the terms, as shown in Table 6. The 4^th ^column of this table lists the absolute number of terms that were associated with multiple gene identifiers. Again we observe that Yeast is the least ambiguous (168 terms and an average of 1.013 identifiers per term, column 5), while Fly, with the most terms on average per gene, is also the most ambiguous, at 1.085 gene identifiers per terms. Again, Fly has the largest standard deviation: only 3.6% of Fly terms are ambiguous – the remaining 96.4 % of Fly terms are associated with a single ID.

**Figure 3:**
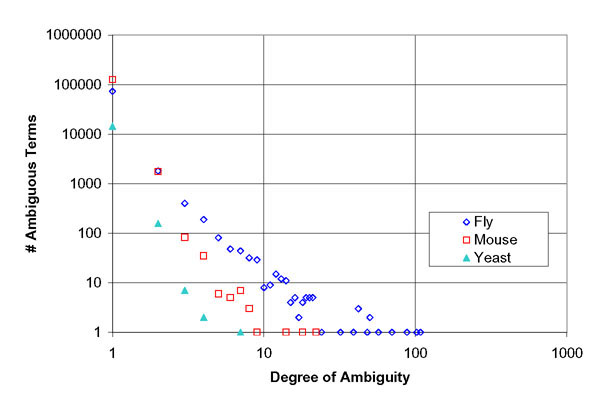
Distribution of ambiguous synonyms in Fly, Mouse and Yeast task 1B lexical resources.

Figure 3 shows the distribution of terms associated with multiple gene identifiers as a log-log plot of number of terms plotted against degree of ambiguity for all three organisms. For degree = 1 (no ambiguity), we see that Mouse has the largest number of terms, then Fly, then Yeast. For degree = 2 (number of terms associated with two gene identifiers), Fly and Mouse are equal; and after that, Fly has by far the most ambiguity, with some terms over 100 ways ambiguous, while Yeast tails off very quickly (one term is 8-ways ambiguous).

In addition, Table 6 shows the ambiguity between gene terms and English vocabulary. The 6^th ^column shows the absolute number of synonyms that overlap with the 5000 most common English words, and the last column shows the average number of ambiguities per synonym (measured against the list of 5000 common words). These numbers are low, but they are also an underestimate of the English ambiguity problem, since some of the ambiguities ("Est" for "esterase-6" or "dorsal" as a gene name) can overlap with specialized biology terminology. Again, we see that there is very little overlap with English for Yeast (2 terms out of 15,000), it is much higher for Mouse (205 out of 53,000 terms) and higher still for Fly (396 terms out of 28,000).

These figures correlate with the differences in difficulty between Yeast, Fly and Mouse. Yeast was relatively easy, with few problems of ambiguity; Fly and Mouse were both significantly harder, for somewhat different reasons. The Fly lexical resources had the most terms per gene ID, and were also the most ambiguous (with respect to gene identifiers and also with respect to overlap with regular English words). Mouse, on the other hand, had longer names and fewer synonyms. This may mean that there were variants of complex names that did not appear in the lexicon, requiring more complex procedures to match gene mention and gene ID. However, this was offset in part by the fact that Mouse had less ambiguity than in Fly. Finally, Mouse had the most noisily annotated training data (recall estimated at 55%), which may have contributed to the difficulty of that task. The top scores for Mouse and Fly were quite similar: for Fly, the high recall was 0.841, precision 0.831 and F-measure of 0.815 (all these scores were from the same group, but not from the same run [7]); for Mouse, high recall was 0.898, precision 0.828, and F-measure 0.791; for Mouse, these three high scores came from three different groups – see Table 4.

## Discussion

There were eight groups participating in task 1B; 7 groups submitted 15 systems for Yeast; 6 groups submitted 11 systems for Fly; and 7 groups submitted 16 systems for Mouse.

Of the eight participating groups, two groups did not submit extended write-ups and are not discussed in detail here. Four systems are documented in articles in this issue [7-10]. For descriptions of the other two systems, see [11,12] in the BioCreAtIvE Workshop Handout [13]. The remainder of this section discusses the challenges presented by task 1B and how the participating systems approached these challenges.

### Technical challenges for Task 1B

The requirements for task 1B can be divided into four steps:

• Identifying gene mentions in the text

• Associating gene mentions to one or more unique gene identifiers

• Selecting the correct gene identifier in cases of ambiguity

• Assembling the final gene list for each abstract

These steps were highly interdependent. There are complex recall/precision trade-offs that occur in capturing candidate gene mentions and in assigning a unique (and correct) gene identifier to these mentions. This is because of significant ambiguity among gene terms (one word might be a term for multiple genes) and also because of significant overlap between gene synonyms ("white", "dorsal") and English vocabulary. For example, the entry for FBgn0000009 consists of the terms "A", "Abnormal" and "Abnormal abdomen". Both "A" and "Abnormal" appear as regular English words (not referring to a gene). Furthermore, there are some 20 other genes that have the term "A" as one of their allowed synonyms. Complicating this further, the term lists provided by the model organism databases, while extensive, were by no means exhaustive. As noted above, the lexical resources differed by organism in number of terms per gene identifier and in ambiguity of terms within the resource.

Precision errors could be caused by:

• False alarms for gene mentions (for example, taking an English word to be a gene name);

• Incorrect disambiguation of ambiguous gene names (which would also cause a recall error);

• Assignment of gene identifiers to genes from non-relevant organisms (e.g., human genes are often discussed in Mouse abstracts, but should *not *be entered into the gene list).

Recall errors could be caused by:

• Failure to recognize a gene mention (perhaps due to mismatch with the organism-specific synonym list)

• Incorrect disambiguation of ambiguous gene names

### Finding gene mentions

The participating groups took a variety of approaches to these challenges. For gene mentions, the approaches fell into roughly two groups:

• Matching against the lexical resource; in many cases, an approximate matching approach was used. For example, [8] used exhaustive pattern matching against the synonym lists to generate a high recall/low precision set of candidates (91% for fly; 79% for mouse; and 90% for Yeast). This was followed by application of a classifier to select the candidates to appear on the final normalized gene list. The approach described in [12] used an enriched lexical resource to achieve high recall (but lower precision) results for Mouse and Yeast.

• Gene mention identification as done for task 1A, adapted to the three specific organisms in 1B [11]. To do this, Hachey et al used a technique to generate "noisy" training data similar to that described in [14].

### Association with unique gene identifier

The second stage, association with a unique identifier, was essentially a table look-up. For groups that used a task 1A-type gene mention tagger, they were then able to use the table look up to filter out erroneous gene mention candidates. However, recall at this step was limited by the completeness of the synonym list from the model organism database. While the term lists contained many variant forms (see the example with *Est-6 *in Figure 1), there were still more variations that had to be handled. The incompleteness of the lexical resources could lead to recall errors.

This was also the stage at which ambiguity was flagged, since some terms could refer to multiple genes (see Table 6). A number of groups chose to edit the lexical resources, removing highly ambiguous or uninformative terms and adding additional variants or descriptions [7-10]. The systematic editing and expansion of the underlying lexical resources was at the core of two high performing systems [7,9]. Both Tamames [10] and Liu [12] used the same tokenization for the lexicon as was used for the gene mention identification; both systems also used stemming to improve the matching between lexicon terms and candidate gene names in the text.

For several groups, the gene mention tagging, gene identifier look-up and disambiguation were interleaved; for example, Hanisch et al [7] accrued evidence during the process of identifying candidate gene mentions that was then used to disambiguate the gene mention to a specific gene identifier. For Tamames [10], these stages were also combined.

### Disambiguation

The next stage, disambiguation for gene synonyms associated with multiple identifiers, turned out to be the most interesting feature of task 1B. The extensive ambiguity of gene names, particularly for Fly and to a lesser extent, for Mouse (see Figure 3), required that systems include techniques for disambiguation. These included pruning the lexicon or accumulating multiple sources of contextual evidence for use in a classifier. Pruning the lexicon was an attractive option, given the highly skewed distribution of ambiguity in both Mouse and Fly. For Mouse, there were 1900 ambiguous terms (out of 126,000 – 1.5%); for Fly, there were 2700 out of 75,000 ambiguous terms (3.6%). Hanisch et al. [7] used a multi-stage process that included correlating abbreviations with their long forms and also a filter for abstracts based on organism specificity. Liu [12] used features derived from rich lexical resources to create feature vectors used in word sense disambiguation. Crim et al. [8] followed their high recall pattern matching system with a maximum entropy classifier trained to distinguish correct matches from bad matches. Hachey et al [11] used information retrieval techniques to associate candidate gene identifiers with term frequencies in a document. They used this to filter gene identifiers for a given abstract, based on similarity to term occurrences associated with the gene identifiers in abstracts from the training data.

### Generating the final gene list

Once these stages were completed, the systems assembled the final gene list for each abstract as output. For some groups, this stage was parameterized in terms of a certainty threshold. Increasing the threshold traded recall for precision, e.g., in [7] and [12]. One group [8] was able to achieve reasonable performance (well above the median of the reported systems) using a single approach across all three organisms, based on high recall pattern matching, followed by a maximum entropy classifier for remove bad matches. Many groups found that it was possible to use much simpler techniques for Yeast than for Mouse or Fly, due to the more tightly constrained nomenclature.

## Conclusion

BioCreAtIvE demonstrated the ability of automated systems to do gene normalization for a range of organisms, given a simple lexical resource consisting of the set of unique gene identifiers and their names and synonyms, and a corpus (5000 abstracts) of noisy training data. The actual performance depended more on the organism than on the kind of system. Factors included the number of genes, the number of synonyms per gene identifier, the consistency of naming conventions, the length and complexity of names, and the degree of ambiguity in the naming conventions. The more ambiguity (among genes, between genes and English) and the more complex the names (descriptions versus simple gene symbols), the harder the problem. Yeast naming is relatively simple and regular – and good performance could be achieved with relatively simple methods (such as expanded lexical look-up). Fly is hard because of ambiguity of short names, both with English words and among gene names; the Flybase lexicon is quite large, with many synonyms per gene; for this task, editing the synonym lists turned out to be a useful technique for reducing ambiguity. Mouse is hard because names are often long and descriptive, subject to many variants (grammatical as well as syntactic and typographic). Mouse was also harder because of our decision to simplify that task to include all gene mentions; this required that the annotators add many genes in by hand, which made training and test data preparation difficult (and somewhat less reliable than other organisms).

Overall, we judged that the BioCreAtIvE task 1B evaluation was a success. We attracted 8 groups from five countries with participation from some of the major groups involved in information extraction in biology. Results demonstrated that the generation of normalized gene lists is well within the range of current technology, although further experiments are needed to determine what performance would be required for a production system used in some semi-automated curation pipeline.

The task raised some interesting research questions:

1. How to achieve high recall – achieving high precision seems relatively easy, but only one system achieved high recall, at the expense of precision [12].

2. How to disambiguate ambiguous synonyms, including both abbreviations or short forms of gene names, and longer forms. This problem requires word sense disambiguation, but this is a new way of framing the problem that should provide an interesting testing ground for various approaches to the problem.

3. How to do rapid adaptation to different task domains, given appropriate lexical resources (synonym list for the organism gene identifiers). Some of the successful systems found that the different organisms benefited from somewhat different approaches. And several systems made use of additional lexical resources. Only one group tried to apply a uniform method across all three organisms [8], with interesting results.

Our approach to using "noisy" training data worked reasonably well, although the noisy data may have imposed limitations on system performance. This reduced the cost of data preparation significantly, but the cost of preparing the training and test sets was greater than we expected: 1–2 person weeks of expert annotator time for a 250 abstract test set. And the difficulties of achieving reliable interannotator agreement were greater than we expected [3]. The training and test data are now available for other groups to use in further experiments.

As we begin to think about a follow on evaluation, the question arises: should this task be repeated? The real task that curators perform uses full text articles (not abstracts, although the Yeast curators do curate from abstracts most of the time). Furthermore, the real task involves a biologically complex set of criteria about which genes to list and which genes that fall outside the scope of what is curated (for example, they belong to another organism, or they are only mentioned in passing). It would be far easier for the organizers to prepare "real" data sets, because it would require none of the editing that was performed for this year's BioCreAtIvE task 1B. On the other hand, it would be harder for the participants, because they would have to handle full text and they would have to replicate biological decisions in terms of which genes to list.

In conclusion, we look forward to receiving feedback from the participants in defining follow-on tasks for the next BioCreAtIvE evaluation.

## Figures and Tables

**Table 1:** Task 1B training and test data sets

**Abstracts**	**Yeast**	**Fly**	**Mouse**
Training (noisy annotation)	5000	5000	5000
Development test (hand corrected)	108	110	250
Blind Test (extensively corrected)	250	250	250

**Table 2:** Task 1B results on Yeast gene list task

**YEAST**	**F-measure**	**Precision**	**Recall**	**True Positives**	**False Positives**	**Missed**
**Gold Standard**	**1.000**	**1.000**	**1.000**	**613**	**0**	**0**
**Noisy Training**	**0.918**	**0.985**	**0.86**	**527**	**8**	**86**

**Hi**	**0.921**	**0.969**	**0.962**	**590**	**329**	**171**
**Low**	**0.763**	**0.642**	**0.721**	**442**	**15**	**23**
**Median**	**0.858**	**0.94**	**0.848**	**520**	**34**	**93**

user5_1B_1	0.819	0.948	0.721	442	24	171
user5_1B_2	0.848	0.915	0.79	484	45	129
user5_1B_3	0.848	0.969	0.754	462	15	151
user6_1B_1	0.857	0.912	0.809	496	48	117
user6_1B_2	0.858	0.907	0.814	499	51	114
user8_1B_1	0.921	0.950	0.894	548	29	65
user8_1B_2	0.910	0.950	0.873	535	28	78
user16_1B_1	0.897	0.951	0.848	520	27	93
user16_1B_2	0.899	0.966	0.840	515	18	98
user16_1B_3	0.897	0.951	0.848	520	27	93
user18_1B_1	0.904	0.94	0.871	534	34	79
user19_1B_1	0.773	0.646	0.962	590	324	23
user19_1B_2	0.77	0.642	0.962	590	329	23
user19_1B_3	0.763	0.661	0.902	553	284	60
user24_1B_1	0.897	0.917	0.878	538	49	75

**Table 3:** Task 1B Results on Fly gene list task

**FLY**	**F-measure**	**Precision**	**Recall**	**True Positives**	**False Positives**	**Missed**
**Gold Standard**	**1.000**	**1.000**	**1.000**	**429**	**0**	**0**
**Noisy Training**	**0.834**	**0.863**	**0.807**	**346**	**55**	**83**

**Hi**	**0.815**	**0.831**	**0.841**	**361**	**684**	**266**
**Low**	**0.284**	**0.224**	**0.380**	**163**	**70**	**68**
**Median**	**0.661**	**0.659**	**0.732**	**314**	**146**	**115**

user5_1B_1	0.661	0.592	0.748	321	221	108
user5_1B_2	0.612	0.659	0.571	245	127	184
user5_1B_3	0.602	0.693	0.531	228	101	201
user8_1B_1	0.665	0.638	0.695	298	169	131
user8_1B_2	0.726	0.692	0.765	328	146	101
user16_1B_1	0.781	0.728	0.841	361	135	68
user16_1B_2	0.815	0.831	0.800	343	70	86
user16_1B_3	0.787	0.744	0.834	358	123	71
user18_1B_1	0.417	0.463	0.380	163	189	266
user19_1B_1	0.284	0.224	0.389	167	580	262
user23_1B_1	0.440	0.315	0.732	314	684	115

**Table 4:** Task 1B results on Mouse gene list task

**MOUSE**	**F-measure**	**Precision**	**Recall**	**True Positives**	**False Positives**	**Missed**
**Gold Standard**	**1.000**	**1.000**	**1.000**	**540**	**0**	**0**
**Noisy Training**	**0.709**	**0.99**	**0.552**	**298**	**3**	**242**

**Hi**	**0.791**	**0.828**	**0.898**	**485**	**674**	**267**
**Low**	**0.571**	**0.418**	**0.506**	**273**	**69**	**55**
**Median**	**0.738**	**0.765**	**0.730**	**394**	**131**	**146**

user5_1B_1	0.672	0.767	0.598	323	98	217
user5_1B_2	0.737	0.811	0.676	365	85	175
user5_1B_3	0.619	0.798	0.506	273	69	267
user6_1B_1	0.739	0.813	0.678	366	84	174
user6_1B_2	0.745	0.785	0.709	383	105	157
user8_1B_1	0.744	0.828	0.676	365	76	175
user8_1B_2	0.661	0.635	0.689	372	214	168
user16_1B_1	0.772	0.750	0.794	429	143	111
user16_1B_2	0.777	0.807	0.750	405	97	135
user16_1B_3	0.791	0.765	0.819	442	136	98
user18_1B_1	0.686	0.728	0.648	350	131	190
user19_1B_1	0.580	0.428	0.898	485	648	55
user19_1B_2	0.571	0.418	0.898	485	674	55
user19_1B_3	0.606	0.489	0.798	431	451	109
user24_1B_1	0.767	0.735	0.802	433	156	107
user24_1B_2	0.776	0.764	0.787	425	131	115

**Table 5:** Lexical Resources: synonymy for Yeast, Mouse, Fly

	**# ID**	**# Terms**	**Terms per ID (St dev)**	**Avg Length (wds) per Term (St dev)**
Yeast	7,928	14,756	1.861 (1.01)	1.001 (0.05)
Mouse	52,594	130,548	2.482 (1.12)	2.772 (2.57)
Fly	27,749	81,711	2.944 (3.88)	1.470 (0.97)

**Table 6:** Lexical resources for Yeast, Fly and Mouse: identifiers, terms, and ambiguity

	**# IDs**	**# Terms**	**Ambiguous Terms**	**Avg # IDs per Term (St dev)**	**# Terms Overlap w English**	**Avg Eng Amb per Term**
Yeast	7,928	14,756	168	1.013 (0.14)	2	0.00014
Mouse	52,594	130,548	1919	1.017 (0.18)	205	0.00171
Fly	27,749	81,711	2736	1.085 (1.03)	396	0.00650

## References

[B1] Yeh AS, Morgan A, Colosimo M, Hirschman L (2005). BioCreAtIvE task 1A: gene mention finding evaluation. BMC Bioinformatics.

[B2] Blaschke C, Leon EA, Krallinger M, Valencia A (2005). Evaluation of BioCreAtIvE assessment of task 2. BMC Bioinformatics.

[B3] Colosimo M, Morgan A, Yeh A, Colombe J, Hirschman L (2005). Data Preparation and Interannotator Agreement: BioCreAtIvE Task 1B. BMC Bioinformatics.

[B4] The FlyBase Database. http://flybase.org/.

[B5] The Mouse Genome Database. http://www.informatics.jax.org.

[B6] Saccharomyces Genome Database. http://www.yeastgenome.org.

[B7] Hanisch D, Fundel K, Mevissen H-T, Zimmer R, Fluck J (2005). ProMiner: Organism-specific protein name detection using approximate string matching. BMC Bioinformatics.

[B8] Crim J, McDonald R, Pereira F (2005). Automatically Annotating Documents with Normalized Gene Lists. BMC Bioinformatics.

[B9] Fundel K, Güttler D, Zimmer R, Apostolakis J (2005). A simple approach for protein name identification: prospects and limits. BMC Bioinformatics.

[B10] Tamames J (2005). Text Detective: Text Dectective: A rule-based system for gene annotation in biomedical texts. BMC Bioinformatics.

[B11] Hachey B, Nguyen H, Nissim M, Alex B, Grover C (2004). Grounding Gene Mentions with Respect to Gene Database Identifiers. BioCreAtIvE Workshop Handouts, Granada, Spain.

[B12] Liu H (2004). BioTagger: A Biological Entity Tagging System. BioCreAtIvE Workshop Handouts, Granada, Spain.

[B13] (2004). [BioCreAtIvE 2004]. BioCreAtIvE Workshop Handouts, Granada, Spain.

[B14] Morgan A, Hirschman L, Colosimo M, Yeh A, Colombe J (2004). Gene Name Identification and Normalization Using a Model Organism Database. J Biomedical Informatics.

